# Impact of CPAP and ventilator system resistance on respiratory effort in healthy volunteers

**DOI:** 10.1186/s12890-026-04649-6

**Published:** 2026-08-28

**Authors:** Simon Lindner, Lena Doerflinger, Luisa S. Drotleff, Burcu Link, Benjamin Neetz, Julia D. Michels-Zetsche, Felix J. F. Herth, Daniel Duerschmied, Simone Britsch

**Affiliations:** 1 Department of Cardiology, Haemostaseology and Medical Intensive Care, Medical Centre Mannheim, Medical Faculty Mannheim, Heidelberg University, Theodor-Kutzer-Ufer 1-3, Mannheim, 68167 Germany; 2 European Center for AngioScience (ECAS), German Center for Cardiovascular Research (DZHK) partner site Heidelberg/ Mannheim, and Centre for Cardiovascular Acute Medicine Mannheim (ZKAM), Medical Centre Mannheim, Heidelberg University, Mannheim, Germany; 3 Department of Pneumology and Critical Care, Translational Lung Research Center Heidelberg (TLRC-H), German Center for Lung Research (DZL), Thoraxklinik Heidelberg, University of Heidelberg, Heidelberg, Germany

**Keywords:** Respiratory Effort, Non-Invasive Ventilation, Continuous Positive Airway Pressure

## Abstract

**Introduction:**

Continuous positive airway pressure (CPAP) is widely used for the treatment of acute and chronic respiratory failure and inspiratory support pressure (ΔP_insp_) can be added to achieve non-invasive ventilation (NIV). We performed a physiological study to determine the effect of CPAP and inspiratory support pressure on respiratory effort measured by esophageal tidal swings in healthy volunteers.

**Methods:**

Esophageal tidal swings were measured in spontaneously breathing, healthy volunteers undergoing 4 airway pressure conditions: atmospheric pressure (A), on a ventilator without any applied pressures (V), CPAP of 5 cmH_2_O, and NIV with PEEP of 5 cmH_2_O and ΔP_insp_ of 5 cmH_2_O during rest, light, and strenuous exercise. The sequence of airway conditions was randomized and the investigator reading the esophageal pressures was blinded.

**Results:**

Thirty-eight individuals were included in the analysis. Esophageal tidal swing during rest was highest when CPAP was applied, compared to A and NIV. During light and strenuous exercise, both V and CPAP increased respiratory effort, compared to A and NIV, suggesting an increase in ventilator system resistance at higher minute ventilation.

**Conclusion:**

CPAP increased respiratory effort compared to both NIV and breathing at atmospheric pressure, but ventilator system resistance appeared to be a more relevant determinant of respiratory effort at higher minute ventilation induced by exercise. As acute respiratory failure often is associated with high minute ventilation, further studies are needed to examine the potential clinical implications of these findings.

## Introduction

Continuous positive airway pressure (CPAP) and noninvasive ventilation (NIV) are cornerstone modalities in the management of acute and chronic respiratory failure, each exerting distinct effects on respiratory mechanics. CPAP delivers a constant level of positive airway pressure throughout the respiratory cycle, primarily acting to increase end-expiratory lung volume by preventing alveolar collapse, reduce airway resistance, and counterbalance lung edema [1–3]. While these effects may lead to a reduction in respiratory effort, active ventilatory support is not provided by CPAP. NIV combines positive airway pressure with additional inspiratory pressure support, thereby actively reducing the work of breathing, increasing tidal volume, and enhancing CO_2_ clearance [4].

Physiological studies have suggested that CPAP could even increase respiratory effort compared to normal breathing and NIV in healthy individuals, as demonstrated by increased diaphragmatic activity and energy expenditure [5, 6]. This effect could be attributed to lung hyperinflation and consequently impaired respiratory mechanics. Additional muscular effort might then be needed to overcome this elevated end-expiratory lung volume, especially when CPAP is applied at higher pressures [5]. Furthermore, the ventilator system itself might introduce restive load, especially at higher minute ventilations [7]. Acute respiratory failure, where NIV is frequently used to avoid intubation, is often accompanied by high minute ventilations [8], rendering this aspect highly relevant for clinical practice. To our knowledge no direct measurements of respiratory effort have been published that would confirm these findings and directly show their impact on non-invasively ventilated humans. Esophageal manometry, used to measure the esophageal tidal swing (ΔP_es_), is one of the most accurate methods to assess respiratory effort in this setting [9]. This technique directly reflects the pressure generated by the inspiratory muscles and provides a reliable and quantitative measure of patient effort [10]. We thus designed this study to investigate the effect of CPAP and NIV on respiratory effort measured by ΔP_es_.

## Materials and methods

### Study design

This study is a randomized and blinded physiological experiment.

### Eligibility criteria

Healthy volunteers aged 18 years or older, capable of providing written informed consent, were recruited for this study. Individuals were excluded from participation if they had a history of bleeding disorders, recurrent bleeding, or current anticoagulant therapy; if they had allergies to ultrasound gel or external materials containing plastic, such as NIV masks or gastric tubes; or if they refused to participate or had medical contraindications to NIV.

### Experimental setup

Participants were seated on a semi-recumbent bicycle ergometer with a 45-degree torso incline throughout the experiment. Prior to the experiment, individual Borg rating of perceived exertion (RPE) scores of 10 (light exercise) and 15 (strenuous exercise) were determined [11]. With a pedaling rate of 40 revolutions per minute, wattage was increased stepwise until the individual’s subjective RPE scores was reached. The corresponding wattages were documented for later use in the experiment. Then, a nasogastric single-balloon catheter for ΔP_es_ measurements was inserted as previously described [12], and a full-face NIV mask was applied and remained in place for the whole duration of the experiment. The experimental phase was initiated when the participant had acclimatized to the presence of nasogastric catheter and NIV mask.

### Test methods

During the experiment, participants underwent different combinations of airway pressures during rest, as well as light and strenuous exercise. The four different applied airway pressure conditions were: no connection to the ventilator, i.e. spontaneous breathing at atmospheric pressure (A), connected to the ventilator but with PEEP and ΔP_insp_ set to 0 cmH_2_O (V), CPAP of 5 cmH_2_O and NIV with a PEEP of 5 cmH_2_O and a ΔP_insp_ of 5 cmH_2_O. The condition V was included to isolate the effect of the ventilator system itself on respiratory effort, independent of applied airway pressure.

The sequence of airway pressure conditions was randomized individually using sealed envelopes. Each combination of airway pressure condition and exercise was undergone until a regular breathing pattern had established, but at least for 2 min. Then, ΔP_es_, airway pressures, respiratory rate, minute ventilation, blood pressure, heart rate, and peripheral oxygen saturation were recorded. The investigators performing the measurements were blinded to the individual setup configuration. Afterwards, the setup was changed according to the randomized protocol until all combinations had been undergone.

### Ventilator and mode

A Carescape™ R860 (GE HealthCare^®^, Chicago, USA) ventilator with dual-limb tubing (separate inspiratory and expiratory line) was used in all participants. Continuous positive airway pressure/pressure support (CPAP/PS) mode with leak compensation activated was used exclusively throughout the experiment.

### Analysis

Statistical analyses were performed using R Version 4.6.0 (R Foundation for Statistical Computing, Vienna, Austria). Komolgorov-Smirnov and Shapiro-Wilk Tests suggested non-normal distribution of ΔP_es_. Metric variables are given as medians and interquartile range (IQR), Qualitative variables are given in absolute numbers and group related percentages. Global differences between measurements were compared using the related-samples Friedman’s two-way analysis of variance by ranks, Friedman’s Q with 3 degrees of freedom along with Kendall’s W (effect size) and 2-sided *p*-value are reported. Post-hoc pairwise comparisons were calculated using pairwise Wilcoxon signed-rank tests, effect sizes (r) were derived from the standardized Wilcoxon test statistic (Z). The legacy Wilcoxon output format was used to extract Z values in a consistent manner across all pairwise comparisons. *P*-values were adjusted by the Bonferroni correction for multiple tests.

No formal sample size determination was possible for this research question, due to the absence of published data on esophageal pressure tidal swings in healthy volunteers undergoing CPAP and NIV during exercise. Similar physiological studies aimed at determining reference values for esophageal manometry have used comparable sample sizes and this sample size offers sufficient precision to detect moderate to large physiological effects, whilst remaining feasible.

## Results

### Participants

Thirty-eight healthy volunteers agreed to participate in this study. Baseline characteristics are displayed in Table 1.

**Table 1:** Baseline characteristics

Number of participants, *n* (%)	38
Age [years], median (IQR)	25 (22–27)
Female sex, *n* (%)	18 (52)
Height [cm], median (IQR)	175 (168–180)
Weight [kg], median (IQR)	76,5 (68–88)
Body mass index [kg/m^2^], median (IQR)	25 (22–27)
Heart rate [bpm], median (IQR)	74 (71–86)

*IQR  *Interquartile range

### Influence of CPAP and NIV on respiratory effort during rest

**Fig. 1:**
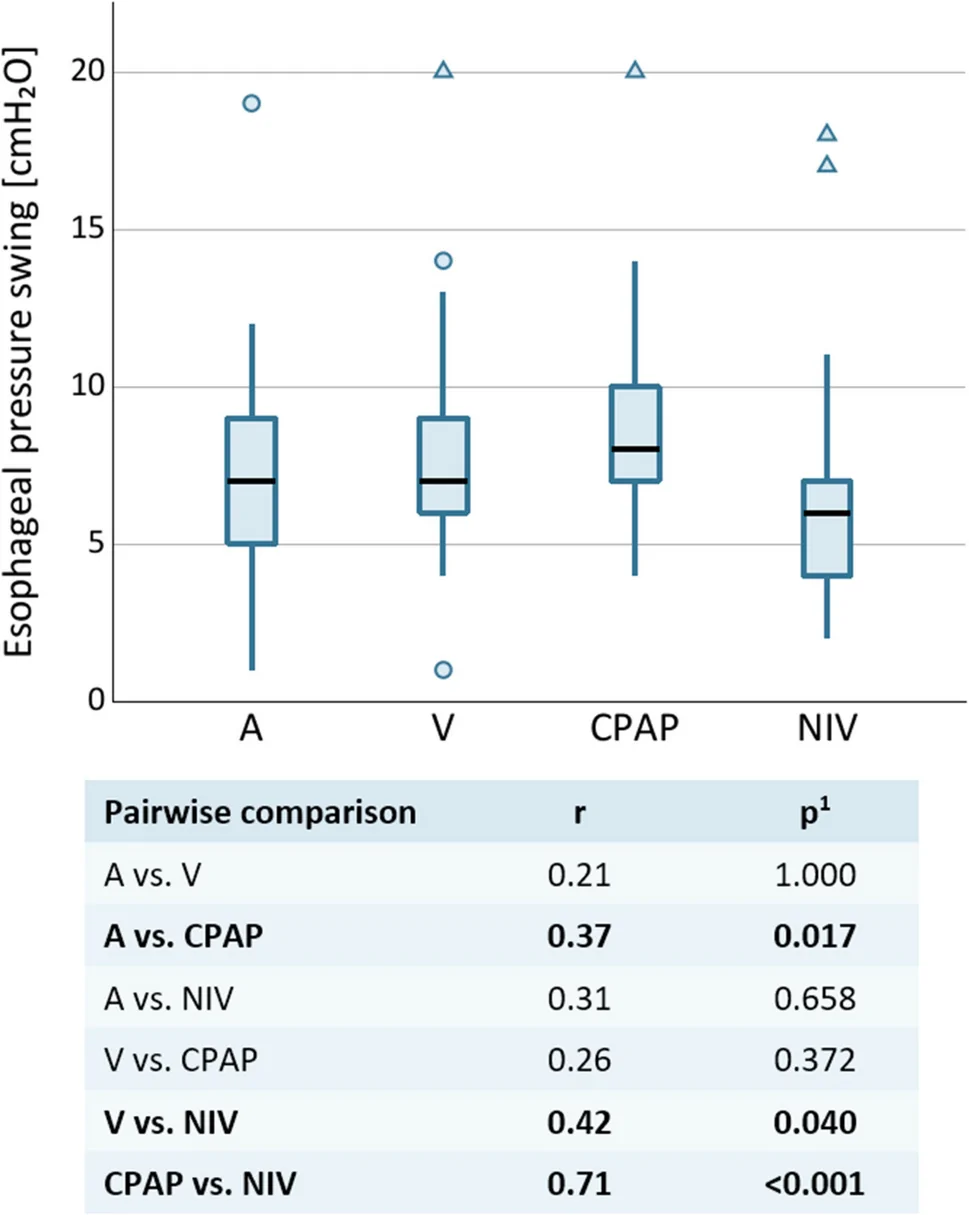
Esophageal pressure swing according to airway pressure conditions during rest. A: atmospheric pressure (no ventilator connection); V: connected to the ventilator system, all pressure settings 0 cmH_2_0, i.e. isolated ventilator-related resistance; CPAP: continuous positive airway pressure of 5 cmH_2_0; NIV: non-invasive ventilation (PEEP 5 cmH_2_0, inspiratory pressure support 5 cmH_2_0). r = effect size. 1: Bonferroni adjusted *p*-value. Global between group difference was calculated as Q = 24.0, W = 0.21, *p* < 0.001

Respiratory effort measured by ΔP_es_ during rest was different between the applied airway pressure conditions (Q = 24.0, W = 0.21, *p* < 0.001). Median ΔP_es_ was highest when CPAP was applied, compared to atmospheric pressure (8 cmH_2_O (IQR 7–10) vs. 7 cmH_2_O (IQR 5–9); *r* = 0.37, *p* = 0.017) and NIV (8 cmH_2_O (IQR 7–10) vs. 6 cmH_2_O (IQR 4–7); *r* = 0.71, *p* < 0.001), see Fig. 1 and Table 2.

**Table 2:** Recorded parameters during rest. A: atmospheric pressure (no ventilator connection); V: connected to the ventilator system; CPAP: continuous positive airway pressure; NIV: non-invasive ventilation; PEEP: positive end-expiratory pressure; ΔPinsp: inspiratory pressure support; ΔPes: tidal change in esophageal pressure; IQR: interquartile range

Airway pressures	A	V	CPAP	NIV
*PEEP [cmH*_*2*_ *O]*	none	0	5	5
*ΔP*_*insp*_ *[cmH*_*2*_*O]*	none	0	0	5
*ΔP*_*es*_ *[cmH*_*2*_*O]*				
Median (IQR)	7 (5–9)	7 (6–9)	8 (7–10)	6 (4–7)
Mean rank	2.3	2.6	3.2	1.8
*p*	< 0.001			
Respiratory rate [bpm]				
Median (IQR)	14 (12–15)	14 (11–15)	12 (10–14)	12 (9–13)
Mean rank	2.9	3.1	2.2	1.9
*p*	< 0.001			
Tidal volume [ml/kg PBW]				
Median (IQR)	-	10 (8–13)	11 (9–15)	15 (12–20)
Mean rank		1.4	1.7	2.8
*p*	< 0.001			
Minute ventilation [l/min]				
Median (IQR)	-	9 (8–11)	9 (8–11)	11 (9–14)
Mean rank		1.7	1.7	2.6
*p*	< 0.001			
SpO_2_ [%]				
Median (IQR)	97 (96–98)	97 (96–98)	98 (97–98)	98 (97–99)
Mean rank	2.2	2.2	2.6	3.0
*p*	0.001			
Mean arterial pressure [mmHg]				
Median (IQR)	96 (90–102)	96 (90–101)	96 (93–102)	95 (91–104)
Mean rank	2.5	2.1	2.8	2.6
*p*	0.117			
Heart rate [bpm]				
Median (IQR)	74 (71–86)	77 (70–85)	77 (70–84)	76 (73–85)
Mean rank	2.4	2.6	2.2	2.8
*p*	0.127			

### Influence of CPAP and NIV on respiratory effort during light exercise

**Fig. 2:**
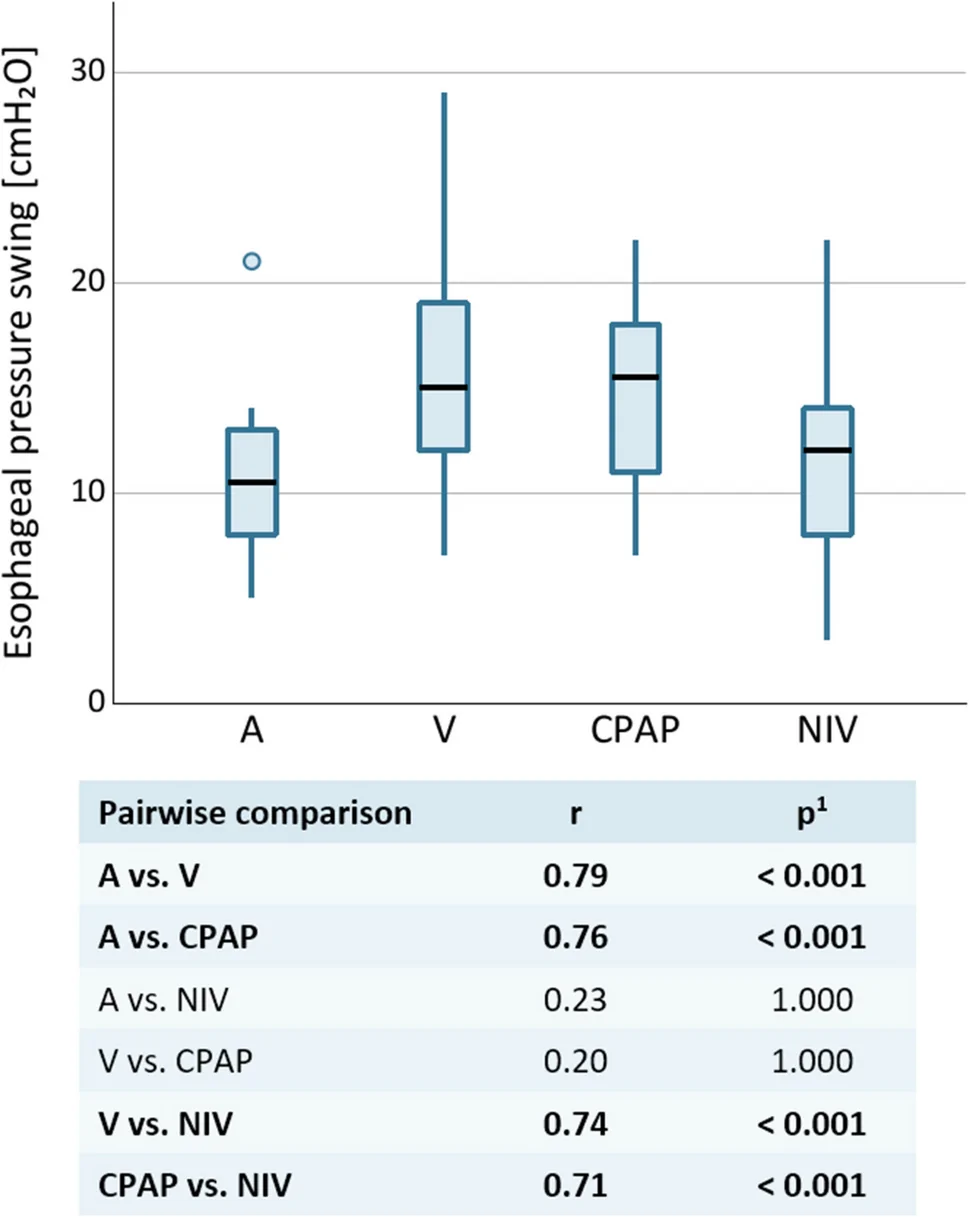
Esophageal pressure swing according to airway pressure conditions during light exercise. A: atmospheric pressure (no ventilator connection); V: connected to the ventilator system, all pressure settings 0 cmH_2_0, i.e. isolated ventilator-related resistance; CPAP: continuous positive airway pressure of 5 cmH_2_0; NIV: non-invasive ventilation (PEEP 5 cmH_2_0, inspiratory pressure support 5 cmH_2_0). r = effect size. 1: Bonferroni adjusted *p*-value. Global between group difference was calculated as Q = 53.7, W = 0.47, *p* < 0.001

Respiratory effort measured by ΔP_es_ when light exercise was performed was different between the applied airway pressure conditions (Q = 53.7, W = 0.47, *p* < 0.001). Respiratory effort undergoing V and CPAP was similar (15 cmH_2_O (IQR 12–19) vs. 16 cmH_2_O (IQR 11–18), *r* = 0.20, *p* = 1.000), and higher than compared to both A and NIV, see Fig. 2 and Table 3.

**Table 3:** Recorded parameters during light exercise. A: atmospheric pressure (no ventilator connection); V: connected to the ventilator system; CPAP: continuous positive airway pressure; NIV: non-invasive ventilation; PEEP: positive end-expiratory pressure; ΔPinsp: inspiratory pressure support; ΔPes: tidal change in esophageal pressure; IQR: interquartile range

Airway pressures	A	V	CPAP	NIV
*PEEP [cmH*_*2*_ *O]*	none	0	5	5
*ΔP*_*insp*_ *[cmH*_*2*_*O]*	none	0	0	5
*ΔP*_*es*_ *[cmH*_*2*_*O]*				
Median (IQR)	11 (8–13)	15 (12–19)	16 (11–18)	12 (8–14)
Mean Rank	1.6	3.4	3.1	1.9
*p*	< 0.001			
Respiratory rate [bpm]				
Median (IQR)	18 (14–21)	15 (13–18)	15 (11–18)	15 (14–18)
Mean rank	3.2	2.4	2.1	2.4
*p*	< 0.001			
Tidal volume [ml/kg PBW]				
Median (IQR)	-	18 (16–21)	20 (16–22)	19 (17–24)
Mean rank		1.8	2.2	2.0
*p*	0.233			
Minute ventilation [l/min]				
Median (IQR)	-	20 (16–22)	19 (17–21)	20 (17–23)
Mean rank		1.9	1.7	2.4
*p*	0.014			
SpO_2_ [%]				
Median (IQR)	96 (95–97)	96 (95–97)	96 (95–96)	96 (95–97)
Mean rank	2.8	2.4	2.3	2.5
*p*	0.278			
Mean arterial pressure [mmHg]				
Median (IQR)	99 (93–107)	100 (93–107)	102 (94–107)	104 (96–108)
Mean rank	2.2	2.4	2.5	2.9
*p*	0.106			
Heart rate [bpm]				
Median (IQR)	101 96–110	102 93 − 11	102 94–108	103 92–109
Mean rank	2.6	2.7	2.2	2.5
*p*	0.431			

### Influence of CPAP and NIV on respiratory effort during strenuous exercise

**Fig. 3:**
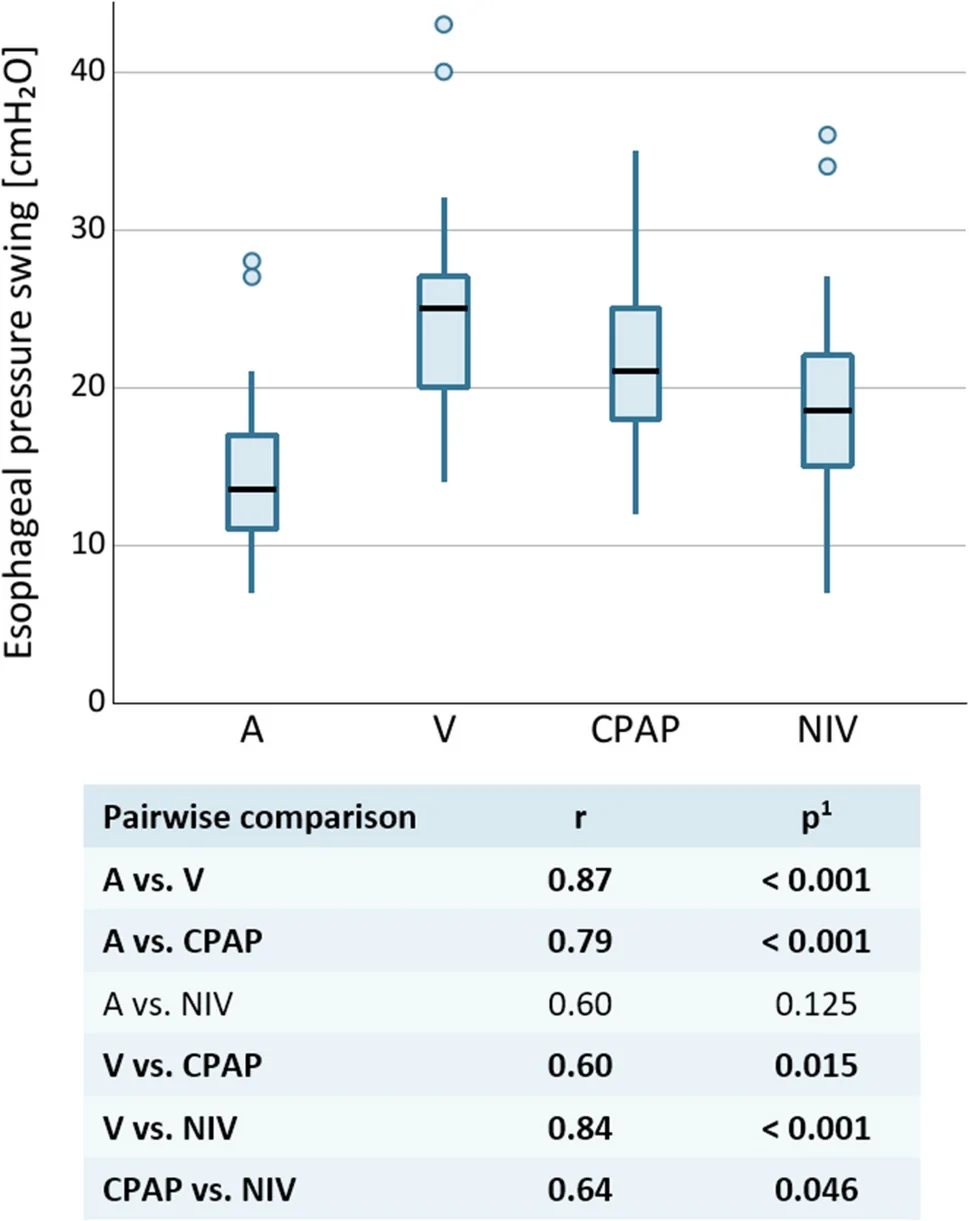
Esophageal pressure swing according to airway pressure conditions during strenuous exercise. A: atmospheric pressure (no ventilator connection); V: connected to the ventilator system, all pressure settings 0 cmH_2_0, i.e. isolated ventilator-related resistance; CPAP: continuous positive airway pressure of 5 cmH_2_0; NIV: non-invasive ventilation (PEEP 5 cmH_2_0, inspiratory pressure support 5 cmH_2_0). r = effect size. 1: Bonferroni adjusted *p*-value. Global between group difference was calculated as Q = 73.6, W = 0.65, *p* < 0.001

Respiratory effort measured by ΔP_es_ performing strenuous exercise was different between the applied airway pressure conditions (Q = 73.6, W = 0.65, *p* < 0.001). Respiratory effort undergoing was highest during V compared to all other pressure conditions. CPAP also had a higher ΔP_es_ compared to A (21 cmH_2_O (IQR 18–25) vs. 14 cmH_2_O (IQR 11–17), *r* = 0.79, *p* < 0.001) and NIV (21 cmH_2_O (IQR 18–25) vs. 19 cmH_2_O (IQR 15–22), *r* = 0.64, *p* = 0.046), see Fig. 3; Table 4.

**Table 4:** Recorded parameters during strenuous exercise. A: atmospheric pressure (no ventilator connection); V: connected to the ventilator system; CPAP: continuous positive airway pressure; NIV: non-invasive ventilation; PEEP: positive end-expiratory pressure; ΔPinsp: inspiratory pressure support; ΔPes: tidal change in esophageal pressure; IQR: interquartile range

Airway pressures	A	V	CPAP	NIV
*PEEP [cmH*_*2*_ *O]*	none	0	5	5
*ΔP*_*insp*_ *[cmH*_*2*_*O]*	none	0	0	5
*ΔP*_*es*_ *[cmH*_*2*_*O]*				
Median (IQR)	14 (11–17)	25 (20–27)	21 (18–25)	19 (15–22)
Mean Rank	1.4	3.7	2.8	2.1
*p*	< 0.001			
Respiratory rate [bpm]				
Median (IQR)	23 (18–26)	18 (16–20)	18 (13–22)	19 (15–22)
Mean rank	3.6	2.2	1.9	2.3
*p*	< 0.001			
Tidal volume [ml/kg PBW]				
Median (IQR)	-	24 (21–28)	25 (23–30)	27 (22–29)
Mean rank		1.7	2.2	2.1
*p*	0.034			
Minute ventilation [l/min]				
Median (IQR)	-	30 (25–35)	31 (25–36)	33 (27–37)
Mean rank		1.6	1.8	2.6
*p*	< 0.001			
SpO_2_ [%]				
Median (IQR)	95 (95–96)	95 (94–96)	95 (94–96)	95 (95–96)
Mean rank	2.6	2.4	2.3	2.6
*p*	0.451			
Mean arterial pressure [mmHg]				
Median (IQR)	105 (95–113)	104 (97–117)	109 (97–118)	109 (99–120)
Mean rank	2.1	2.5	2.9	2.6
*p*	0.112			
Heart rate [bpm]				
Median (IQR)	129 (122–135)	132 (120–138)	127 (120–136)	130 (120–142)
Mean rank	2.0	2.9	2.5	2.6
*p*	0.024			

## Discussion

This physiological study assessed changes in respiratory effort induced by different modes of respiratory support in healthy volunteers using ΔP_es_. We found that CPAP increased respiratory effort compared to both breathing at atmospheric pressure and NIV during rest and all exercise intensities, while connection to the ventilator system was the main determinant of respiratory effort during exercise.

Our results suggest that, at least in healthy lungs, the connection to a mechanical respirator bears the potential to increase respiratory effort, similar to findings in previous experiments [5, 6]. This might be partially attributable to CPAP-induced increases in end-expiratory lung volume [5], potentially resulting in a less efficient force generation due to an altered length–tension relationship of the diaphragm and other inspiratory muscles, as well as sub-optimal lung compliance [13]. As CPAP does not provide active inspiratory support, a resulting additional pressure load would have to be compensated by the subject, increasing necessary muscle effort. This could be specific to healthy subjects with normal lung compliance and may not translate to patients with recruitable or edematous lungs in acute respiratory failure. Firstly, in acute respiratory failure, opening of collapsed alveoli by CPAP may lead to an optimization of respiratory system compliance which lowers the mechanical work of breathing. Secondly, an improved gas exchange achieved by CPAP can subsequently reduce the respiratory drive. However, overinflation due to inadequately high CPAP levels could potentially occur in acute respiratory failure as well [14]. Further research is needed into the effects of CPAP in acute respiratory failure and how these might vary with dosage.

When the work of breathing was artificially increased through introduction of exercise, respiratory effort increased disproportionately when participants were connected to the ventilator, even without applied airway pressures. This suggests an additional resistance might have been induced by the ventilator system, appearing to become relevant at higher rates of minute ventilation, but not during rest. It has been previously demonstrated that at a minute ventilation of 8 L/min and a PEEP of 5 cmH_2_O, the ventilator used in our experiment exhibits a median instantaneous expiratory resistance of 20.9 cmH_2_O/L/s [7]. During rest, where minute ventilation was approximately 10 L/min in our participants, no increased respiratory effort associated with the ventilator system was noted. However, expiratory resistance in ventilator systems increases nonlinearly with flow, as resistance is calculated by the ratio of pressure drop to flow, and the pressure drop across the valve rises disproportionately at higher flows [15]. Extrapolating from the data reported for the ventilator that was used in our experiment, at minute ventilations of 20 and 30 L/min, as achieved during light and hard exercise in our experiment, ventilator system resistance likely exceeded clinically relevant thresholds and increased the work of breathing for our participants. During strenuous exercise, respiratory effort was highest when connected to the ventilator system compared to all other airway pressure conditions.

This might be particularly relevant in acute respiratory failure, where minimizing additional resistive loads is critical to avoid excessive respiratory muscle workload and potentially associated complications such as damage to the lung referred to as patient self-inflicted lung injury (P-SILI) [16, 17]. High minute ventilations ranging from 12 to up to 29 L/min have been reported in those patients [8, 18, 19], rendering ventilator system resistance an additional potentially important determinant of respiratory effort.

The ventilator used in this study is a high-performance, compressed-gas intensive care unit ventilator (Carescape R860, GE Healthcare). Performance among ventilators typically varies regarding trigger delay, pressurization capacity, the accuracy of delivered versus set airway pressures, and expiratory resistance [20]. In the context of NIV, the device’s ability to compensate for leaks is an additional factor of clinical relevance [21]. Regarding the expiratory valve performance of the R860, previous bench studies comparing it to six other ventilators identified a relatively long opening time of 0.110 s and a comparatively high median expiratory resistance of 20.9 cmH_2_O/L/s. However, the device’s ability to control expiratory resistance remains high [7]. Furthermore, the R860 exhibits low error and high precision regarding pressurization capacity [22]. In summary, the ventilator used provides robust pressurization but is characterized by a relatively high internal expiratory resistance. It has been demonstrated, that technical performances vary considerably across ICU ventilators [20]. Further research is needed to unfold the potential clinical consequences of these differences, especially in high minute ventilation states.

As gastric pressures were not measured in this study, it is not possible to determine the extent to which the observed increases in ΔP_es_ are attributable to forced expiration. Active expiration elevates end-expiratory esophageal pressure, which consequently increases the measured ΔP_es_ [23]. Therefore, the high ΔP_es_ values recorded during mild and hard exercise may be at least partially explained by the recruitment of expiratory muscles. Given that expiratory muscle activity is physiologically inevitable at higher metabolic demands, the application of purely inspiratory pressure support is limited in its ability to reduce the total work of breathing under these conditions. The main limitation of this physiological study is that measurements were performed in healthy, young subjects of normal weight and the relatively small sample size. No direct conclusions should be drawn for clinical practice and the treatment of patients.

## Conclusion

The results of this randomized and blinded physiological experiment suggest that while CPAP might negatively impact respiratory mechanics in individuals with healthy lungs, at higher minute volumes induced by exercise, the resistance of the ventilator system plays a much greater role in influencing respiratory effort. As acute respiratory failure often is accommodated by high minute ventilation, future research should investigate these phenomena comparing different ventilator models before direct conclusions in patients receiving CPAP and NIV can be drawn.

## Data Availability

The data that support the findings of this study are not publicly available due to privacy reasons. Anonymized data are available from the corresponding author upon reasonable request.

## Abbreviations

A: atmospheric pressure
CPAP: Continuous positive airway pressure
ΔP_es_: esophageal tidal swing
ΔP_insp_: inspiratory support pressure
NIV: Non-invasive ventilation
PEEP: Positive end-expiratory pressure
V: On a ventilator without any applied pressures
